# Contextual Cueing Accelerated and Enhanced by Monetary Reward: Evidence From Event-Related Brain Potentials

**DOI:** 10.3389/fnhum.2021.623931

**Published:** 2021-04-15

**Authors:** Guang Zhao, Qian Zhuang, Jie Ma, Shen Tu, Shiyi Li

**Affiliations:** ^1^Key Research Base of Humanities and Social Sciences of the Ministry of Education, Academy of Psychology and Behavior, Tianjin Normal University, Tianjin, China; ^2^Faculty of Psychology, Tianjin Normal University, Tianjin, China; ^3^Tianjin Social Science Laboratory of Students’ Mental Development and Learning, Tianjin, China; ^4^Research Center of Brain and Cognitive Neuroscience, Liaoning Normal University, Dalian, China; ^5^Guizhou University of Finance and Economics, Guiyang, China

**Keywords:** contextual cueing, monetary reward, SPN, attentional guidance, visual search

## Abstract

The vital role of reward in guiding visual attention has been supported by previous literatures. Here, we examined the motivational impact of monetary reward feedback stimuli on visual attention selection using an event-related potential (ERP) component called stimulus-preceding negativity (SPN) and a standard contextual cueing (CC) paradigm. It has been proposed that SPN reflects affective and motivational processing. We focused on whether incidentally learned context knowledge could be affected by reward. Both behavior and brain data demonstrated that contexts followed by reward feedback not only gave rise to faster implicit learning but also obtained a larger CC effect.

## Introduction

Our eyes receive a myriad of perceptual inputs at any given moment. Our visual system, however, is severely limited in processing this information (Simons and Rensink, 2005). Thus, there should be some powerful and sophisticated selection mechanisms to focus attention toward objects or events that are most relevant to us. These selection mechanisms are affected by our ability to be sensitive to and take advantage of regularities in the environment. That is, we could use these learned environmental regularities to guide our behavior.

One example of this can be seen in the contextual cueing (CC) effect, which was introduced by Chun and Jiang (1998). The CC effect facilitates search performance for repeated configurations. That is, there is faster search time for consistent spatial contexts as compared with the search times where spatial contexts are varied (Chun and Jiang, 1998, 1999). In their experiments, participants were asked to search for a rotated “T” target embedded in rotated “L” distractors. Configuration of a trial was defined by the spatial layout formed by all the items on that trial, and there were two types of configurations: in one type of configuration, the locations of distractors remained constant across blocks, i.e., the repeated configurations; while in the other type, distractors’ locations varied across blocks, i.e., the novel configurations. They found faster search times in repeated configurations than in novel ones. CC emerges quickly, after about 5 to 10 repetitions; and participants’ performance approaches asymptote after being exposed approximately 30 times to repeated displays. Furthermore, in both repeated and novel configurations, the location of the target remained constant, and the eccentricity of the target location was balanced across configurations.

Thus, the probability of a target appearing in any given location is equated in repeated and novel conditions. This manipulation excludes the possibility of target location probability cueing. Additionally, this effect usually occurs without evidence of conscious memory (Chun and Jiang, 1998, 2003; Greene et al., 2007); thus, CC is usually thought to result from implicit learning (Chun and Jiang, 1998, 1999; Goujon and Fagot, 2013; Zellin et al., 2014), though several studies (Smyth and Shanks, 2008; Geyer et al., 2010, 2012; Schlagbauer et al., 2012; Vadillo et al., 2016) pointed out that this may due to the limited number of repeated displays in the recognition test as compared with the learning phase and declared the explicitness of CC memories (Annac et al., 2019).

Chun and Jiang suggested that CC occurs because the implicitly learned association between the target location and the configuration constrains what to expect and guides attention. That is, the global visual context may implicitly guide spatial attention directly toward the location of the target embedded among the distractor stimuli (Chun and Jiang, 1998, 1999; van Asselen and Castelo-Branco, 2009; Manelis and Reder, 2012; Kasper et al., 2015), which has been supported by convergent evidence from behavioral studies (Manginelli and Pollmann, 2009; Manelis and Reder, 2012) and eye-tracking studies (Manginelli and Pollmann, 2009; van Asselen et al., 2011; Zhao et al., 2012).

A growing body of evidence has pointed to another relevant factor, reward, which affects attention control (Anderson, 2013, 2016a, b, 2017; Chelazzi et al., 2013). Reward learning modifies the attentional priority of stimuli, allowing these stimuli to compete more effectively for selection (Anderson, 2013). For example, evidence from the pop-out visual search task (Kristjánsson et al., 2010) and the color-naming Stroop task (Krebs et al., 2010) has verified that the prospect of a reward enhances the processing of task-relevant stimulus information by increasing participant’s motivation. Furthermore, the evidence from event-related potentials (ERPs) and functional magnetic resonance imaging (fMRI) studies has shown that targets had rewards associated with them, elicited earlier and larger N2pc components, and increased larger activity within cortices reflecting attention resource allocation, suggesting that the deployment of attention is modulated by motivation, i.e., biased toward reward-associated targets (Kiss et al., 2009; Krebs et al., 2012).

Given that a complex set of distractor configuration or a subset thereof is processed and learned as a whole in the search task of a CC paradigm (Olson and Chun, 2002; Kunar et al., 2006; Brady and Chun, 2007; Zhao et al., 2012; Beesley et al., 2014), we expect to extend the reward effect to CC. We were interested in whether rewarding spatial displays could alter the implicit learning process that gives rise to the CC effect: if participants are unaware that they are searching for a repeated configuration, yet they are rewarded at the end of the trial, does this reward change the way participants implicitly learn the repeated context? Specially, we hypothesized that the guidance observed from contexts that consistently trigger a higher reward would be stronger than the guidance observed from contexts that trigger a lower reward or that never trigger any reward because the rewarding context will give participants a higher motivation to accomplish a search task.

However, there are divergent views of whether reward effects on CC were driven by the reward motivation or merely by the target location itself. Tseng and Lleras (2013) found that contextual learning is greatly sensitive to the reward of context–outcome associations: the authors introduced different levels of reward – namely, gaining points (reward condition), losing points (penalty condition), or no reward (no-outcome condition) – into the CC paradigm. These three conditions were present in repeated as well as novel displays. They observed accelerated learning in the reward condition and a CC effect after a single exposure. However, they found that the overall size of the CC effect does not increase by reward. Using an fMRI study, Pollmann et al. (2016) replicated this finding. That is, previously highly rewarded displays are searched more efficiently, which is reflected by selectively less involvement of the dorsal attention network, which is involved in overt and covert attention shifts, even days later and in the absence of reward.

However, Schlagbauer et al. (2014) pointed out an alternative explanation for reward modulation. As we deliberated before, besides the distractor context recurring in repeated displays, the target is also recurring at the very same location, and this is the same in novel displays as well; this offers an opportunity for the reward to modulate target location probability cueing. Tseng and Lleras averaged across the three novel reward conditions at baseline and compared these averaged data with each repeated reward condition. Schlagbauer et al. indicated that this operation did not isolate the effect of reward on the learning of distractor contexts, leading to confusion for the target probability learning. Though they obtained a CC effect with a monetary reward, it is unclear whether the association between reward and target locations in novel displays will still come forth as in repeated displays. Tseng and Lleras had associated reward values consistently with target locations in novel displays in the same way as in repeated displays. In their experiment using the same paradigm as Tseng and Lleras, Schlagbauer and colleagues found evidence for a reward regulation of probability cueing rather than of CC. Recently, Sharifian et al. (2017) proposed an overshadowing hypothesis: rewards become associated with the target location only in new displays, but not in repeated displays, where the repeated target location is overshadowed by the more salient repeated target–distractor configuration. Nevertheless, they emphasized that at the early stage (particularly over the first several repetitions), target location and distractor context will compete for reward association, whereas in the long run, reward association with repeated context dominates.

One way to test whether CC is modulated by reward is to measure the brain activity while participants are performing a CC task. So far, there has not been any electroencephalography (EEG) study performed on healthy human subjects showing that implicit learning could be modulated by reward. In the present study, we recorded the electrophysiological brain activity in addition to the behavioral data. We focus on an ERP component named stimulus-preceding negativity (SPN), which has been proved to be sensitive to the feedback or outcome of one’s task performance in previous experiments. SPN is a slow, right-hemisphere-dominant, negative non-motoric slow potential, which starts a few seconds before feedback onset and exhibits peak amplitudes just before the stimulus presentation, and is thought to index activity in the insula cortex (Brunia, 1988; Brunia and Damen, 1988; Brunia et al., 2011; Zheng and Liu, 2015). The functional significance of SPN has been discussed in terms of emotional anticipation (Michalowski et al., 2015), and a number of recent studies have determined that the SPN is more negative when participants await reward-related feedback (Foti and Hajcak, 2012; Fuentemilla et al., 2013; Morís et al., 2013). If a reward appears after search display, it can affect search times, and we would infer that the participant might have learned the association between reward and search display. Thus, the rewarded display’s subsequent exposure should induce a larger negative SPN amplitude than a non-rewarded display, because of the anticipated reward.

We used the typical CC paradigm introduced by Chun and Jiang (1998). During the experiment, reward values (Reward and Non-reward) were associated with specific target locations for repeated configurations as well as novel configurations. Specifically, there were four kinds of conditions in our experiment, i.e., Repeated-Reward, Repeated-Non-reward, Novel-Reward, and Novel-Non-reward. Each condition consistently corresponded to a search display. Participants’ response was followed by a feedback, informing them if there was monetary reward (Reward condition) or not (Non-reward condition). To rule out the target probability learning, the target locations were evenly distributed throughout the experiment and considering quadrants. Half of the target locations were used for repeated configurations and the other half for new configurations. In this way, reward modulation to CC could be assessed by the interaction of configuration (Repeated, Novel) and reward condition (Reward, Non-reward) on both response time (RT) and SPN amplitudes. We hypothesized that if reward learning regulates the CC, the amplitude of SPN elicited by the repeated-reward trial will be larger than the repeated-non-reward trial; additionally, RT on the repeated-reward trial will be faster than on repeated-non-reward trial.

## Results

### Behavioral Results

#### Accuracy

**FIGURE 1:**
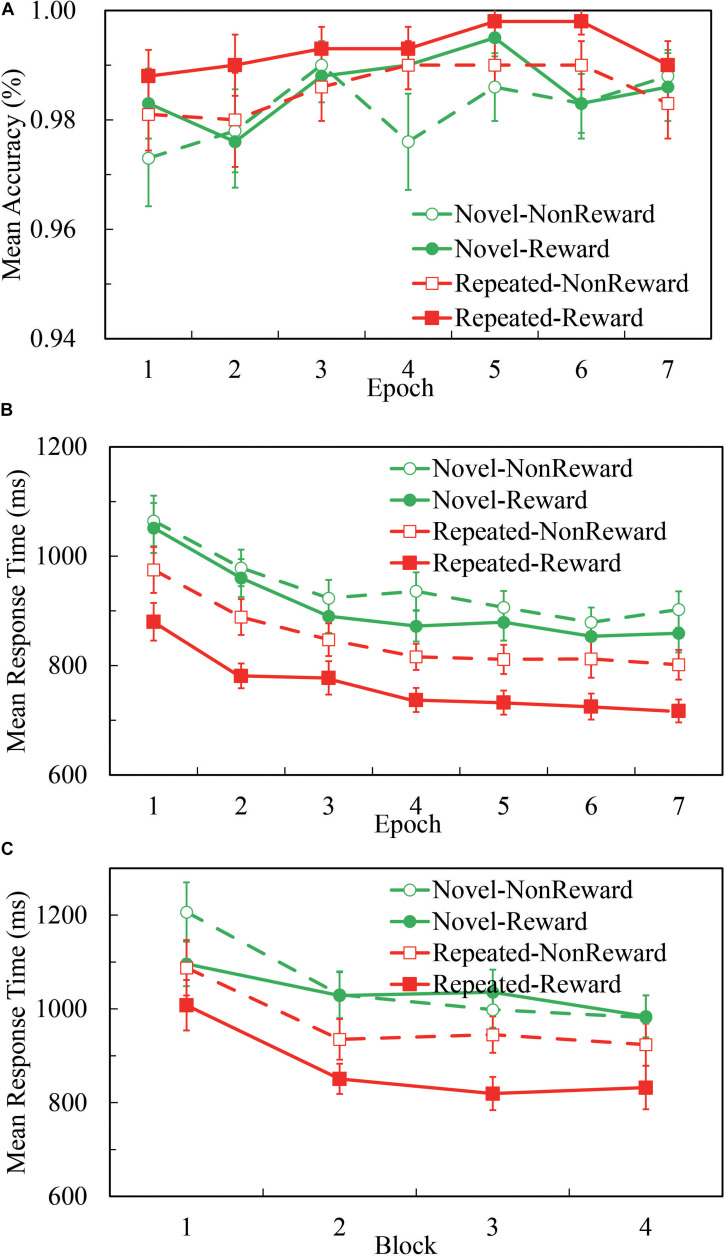
Accuracies and response time (RT) performance in the Repeated-Reward, Repeated-Non-reward, Novel-Reward, and Novel-Non-reward conditions. **(A)** Mean accuracies as a function of epoch (seven epochs, separately averaged in four blocks). Error bars represent the standard error of the mean. **(B)** Mean correct RTs plotted as a function of epoch. Error bars represent the standard error of the mean. **(C)** Mean correct RTs as a function of the block in the first epoch. Error bars represent the standard error of the mean.

Overall, participants’ performance was highly accurate. As Figure 1A illustrates, the mean accuracy was 98.5% for all the four conditions, and 98.5, 99.2, 98.0, and 98.4% for Repeated-Non-reward, Repeated-Reward, Novel-Non-reward, and Novel-Reward configurations, respectively. A 2 (Configuration: Repeated vs. Novel) × 2 (Reward condition: Reward vs. Non-reward) × 7 (Epoch: 1 to 7) repeated-measures ANOVA was performed. The main effects of configuration, *F*(1,24) = 5.510, *p* = 0.027, η^2^ = 0.187; epoch, *F*(1,24) = 2.983, *p* = 0.009, η^2^ = 0.111; and reward, *F*(1,24) = 6.732, *p* = 0.016, η^2^ = 0.219 were significant, with higher accuracy in repeated displays than in novel displays, greater accuracy for the Reward than Non-reward condition, and accuracies increasing over time. No other interaction differences were found (all *p*s > 0.522). This pattern is in accordance with the search time results; thus, there was no speed-accuracy trade-off here.

#### Search Times

A 2 (Configuration: Repeated vs. Novel) × 2 (Reward Condition: Reward vs. Non-reward) × 7 (Epoch: 1 to 7) repeated-measures ANOVA was performed. As Figure 1B illustrates, the main effect of configuration was significant, *F*(1,24) = 78.270, *p* < 0.001, η^2^ = 0.765, about 118-ms search time benefit in repeated displays than in novel displays. The main effect of epoch was significant, *F*(6,144) = 29.723, *p* < 0.001, η^2^ = 0.553, and RTs decreased over time. The main effect of reward was significant, *F*(1,24) = 39.955, *p* < 0.001, η^2^ = 0.625, about 59 ms faster response in the reward condition than non-reward. The interaction between configuration and reward was significant, *F*(1,24) = 7.729, *p* = 0.010, η^2^ = 0.244. The other interactions were not significant (all *p*s ≥ 0.117). *Post hoc* comparisons revealed that RTs for the repeated configuration were significantly smaller than the novel configuration in both the Reward (764.4 ± 25.3 vs. 909.5 ± 34.2 ms, *p* < 0.001) and Non-reward conditions (850.4 ± 31.1 vs. 941.6 ± 34.0 ms, *p* < 0.001); RTs for the Reward condition were significantly greater than the Non-reward condition in both repeated configurations (*p* < 0.001) and novel configurations (*p* < 0.021). Paired *t*-test showed that the difference between repeated and novel configurations is larger for Reward than Non-reward, *t*(24) = 2.780, *p* < 0.010.

Although the interaction of configuration × epoch missed significance: *F*(6,144) = 1.735, *p* = 0.117, η^2^ = 0.067, BF01 = 68.92, we referred to previous studies (Ogawa et al., 2007; Zhao et al., 2012) and split the first epoch into four blocks (see Figure 1C). The 2 (Configuration: Repeated vs. Novel) × 2 (Reward Condition: Reward vs. Non-reward) × 4 (block: 1 to 4) repeated-measures ANOVA analysis showed a significant main effect of configuration, *F*(1,24) = 36.622, *p* < 0.001, η^2^ = 0.604; block, *F*(3,72) = 21.664, *p* < 0.001, η^2^ = 0.474; and reward, *F*(1,24) = 7.017, *p* < 0.014, η^2^ = 0.226. The three-way interaction was marginally significant, *F*(3,72) = 2.641, *p* = 0.056, η^2^ = 0.099. *Post hoc* comparisons of mean RTs revealed a significant configuration effect that appeared as early as in the second block when there was the reward feedback (*p*-values: 0.056, < 0.001, < 0.001, and < 0.001, respectively), while in the Non-reward condition, no statistical differences of configuration were found except in the first block (*p*-values: 0.018, 0.057, 0.384, and 0.086, respectively). The results showed that the CC effect emerged in the first epoch, only after four repetitions of configurations.

Combined with the results of the above two statistical analyses, we can conclude that the CC effect emerged as early as in the first epoch by the rapid configuration learning. We replicated the CC effect and found that rewarding context speeds up the search performance for repeated configurations in visual search.

#### Recognition Test

Similar to previous studies, the knowledge about repeated configurations was implicit. In the recognition test, for the Reward trials, the hit rate (45.3%) did not differ significantly from the false alarm (FA) rate (47.3%), *t*(24) = −0.327, *p* = 0.746, BF01 = 4.516, as well as for the Non-reward condition (hit rate: 57.3%, FA rate: 50.0%), *t*(24) = 1.333, *p* = 0.195, BF01 = 2.158. Thus, no evidence was found for the relation between a possible explicit knowledge about the configurations and CC. Furthermore, there was no significant difference between the two hit rates of the Reward and Non-reward conditions, *t*(24) = −1.953, *p* = −0.063, BF01 = 0.930, or the two FA rates: *t*(24) = −0.473, *p* = 0.640, BF01 = 4.282, showing that reward assignment did not affect the recognition of configurations. Six of 25 participants reported that they noticed that certain configurations were being repeated, but their recognition performance did not differ from the whole group.

### Electrophysiological Results

**FIGURE 2:**
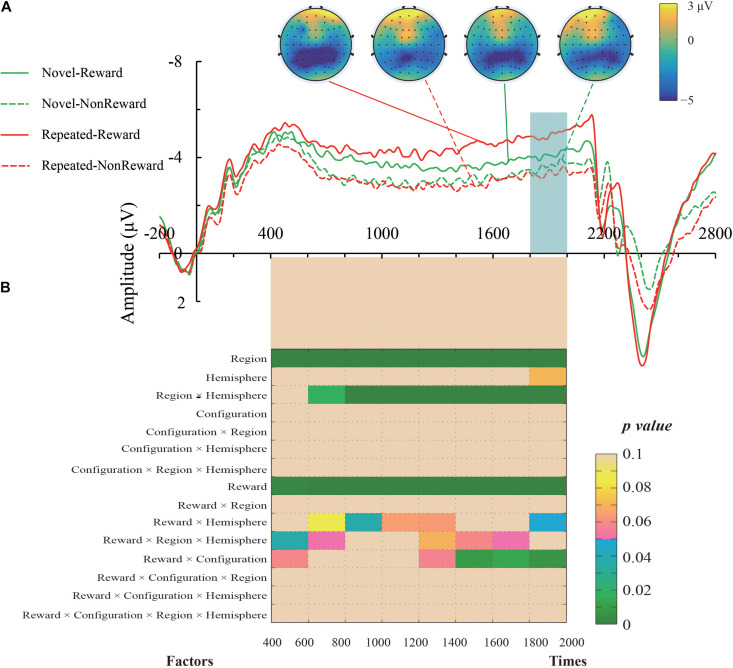
**(A)** Grand average event-related potentials (ERPs) elicited by the anticipation of feedback for each configuration and the reward condition. The stimulus-preceding negativity (SPN) components, which are highlighted by a light gray box, were ranged from 1,800 to 2,000 ms after the anticipants’ response (i.e., 200 to 0 ms before the feedbacks’ appearance). The topographic maps from left to right were the Repeated-Reward, Repeated-Non-reward, Novel-Reward, and Novel-Non-reward conditions at 190 ms before the feedback onset. **(B)** The statistical *p*-values of SPN mean amplitudes were analyzed every 200-ms interval from 400 to 2,000 ms after response onset. Four within-subject factors of repeated-measures ANOVAs designed for Configuration (Repeated/Novel), Reward (Reward/Non-reward), Region (central, centroparietal, parietal), and Hemisphere (left/right).

**FIGURE 3:**
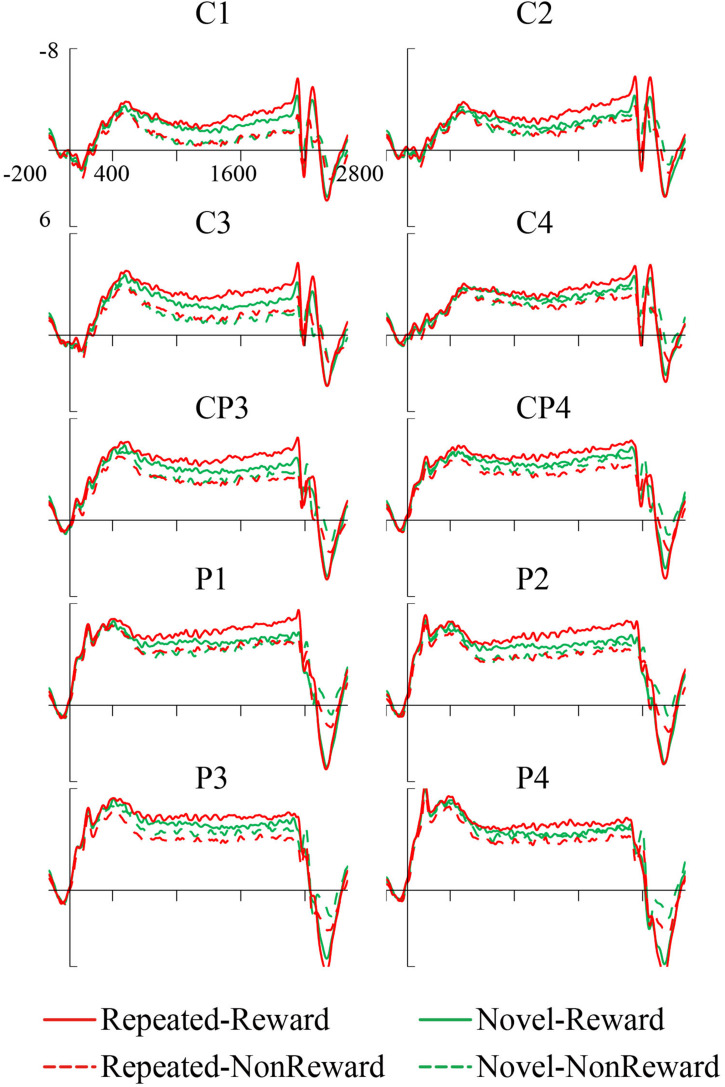
Grand average stimulus-preceding negativity (SPN) event-related potentials (ERPs) to feedback stimuli for each configuration and reward condition at C1, C2, C3, C4, CP3, CP4, P1, P2, P3, and P4.

The mean amplitude of the SPN was measured from 1,800 to 2,000 ms, after the response onset, that is, between -200 and 0 ms prior to feedback onset, as defined in previous research. Central (C1/2, C3/4), centroparietal (CP3/4), and parietal (P1/2, P3/4) electrodes, which are typically maximum amplitude regions for SPN, were analyzed (Pornpattananangkul and Nusslock, 2015). Figure 2A shows the grand averaged SPNs, and Figure 3 shows the separate SPNs of 10 electrode sites for all four conditions. SPNs were clearly present before the feedback stimulus, and for both conditions developed gradually over these regions.

The mean amplitudes of SPN were analyzed with a 2 (Configuration: Repeated/Novel) × 2 (Reward Condition: Reward/Non-reward) × 3 (Region: central, centroparietal, parietal) × 2 (Hemisphere: left/right)repeated-measures ANOVA. The main effect of the reward condition was significant, *F*(1,24) = 17.34, *p* < 0.001, η^2^ = 0.420, showing that the SPN was greater preceding a reward feedback than preceding a blank screen feedback. The main effect of region, *F*(2,48) = 26.52, *p* < 0.001, η^2^ = 0.525, and the interaction of configuration × reward, *F*(1,24) = 8.60, *p* = 0.007, η^2^ = 0.264, were significant. Subsequent simple effects test demonstrated that the SPN amplitude was larger for repeated configuration than novel configuration when there was the reward feedback, *p* < 0.001, whereas no such significant contextual difference was found in the Non-reward condition, *p* = 0.50. The interaction of hemisphere × reward was significant, *F*(1,24) = 4.37, *p* = 0.047, η^2^ = 0.154. Subsequent simple effects test demonstrated that the SPN amplitude was higher on the left hemisphere than the right hemisphere when there was the reward feedback, *p* = 0.011; whereas no such significant cueing difference was found in the Non-reward condition, *p* = 0.307. The interaction of region × hemisphere was significant, *F*(2,48) = 11.47, *p* < 0.001, η^2^ = 0.323. Subsequent simple effects test demonstrated that the left hemisphere preponderances of SPN amplitude were found in the central, *p* = 0.003, and centroparietal regions, *p* = 0.013. The other main effects and interactions were not significant (all *F*s < 1, *p*s > 0.05).

It can be seen that the sustained negativity starts much earlier (at ∼500–600 ms). To further explore the reward expectations on the early stage right before the feedback onset, we entered Configuration (Repeated/Novel), Reward (Reward/Non-reward), Region (central, centroparietal, parietal), and Hemisphere (left/right) into four-factor repeated-measures ANOVAs every 200-ms interval from 400 to 2,000 ms after response onset. The results are plotted in Figure 2B. In general, the SPN exhibited roughly the same electrophysiological pattern as the above results of the 1,800–2,000 ms time window. Specifically, the main effects of Reward and Region were significant throughout the time course. The region × hemisphere interactions were significant right 600 ms after response onset. In the late phase, about 1,400 ms after response activation, the reward × configuration interactions started to emerge. The reward × hemisphere and reward × region × hemisphere interactions were scattered on either side of the time course.

Furthermore, to seek confirmation of Sharifian’s overshadowing hypothesis, which emphasized that at the early stage (particularly over the first several repetitions) target location and distractor context will compete for reward association, whereas in the long run reward association with repeated context dominates, we also compared the SPN amplitudes in the first two epochs for rewarded vs. non-rewarded new displays considering Bayes factor analysis. We found no significant difference of SPN amplitude between epoch 1 and epoch 2 for rewarded [*t*(24) = 0.975, *p* = 0.340, *d* = 0.199, BF01 = 4.672] and for non-rewarded [*t*(24) = 1.070, *p* = 0.296, *d* = 0.218, BF01 = 3.932] new displays. In consideration of the small number of trials in each condition (16 trials), this result may be due to the low analysis power.

## Discussion

The aim of the present study was to investigate whether attentional guidance in the CC paradigm is influenced by monetary reward. Participants’ electrophysiological brain activities and response times were recorded while they were performing a typical CC task with feedback containing reward information. For the first time, we introduced an ERP component called SPN, known to reflect emotional anticipation, specifically reward anticipation (Brunia et al., 2011), to examine whether reward could modulate attention in the CC paradigm. Here, we replicated a recent finding that reward modulates the CC effect by associating repeated configuration as well as target location with reward during the search task (Tseng and Lleras, 2013; Pollmann et al., 2016). We manipulated the outcomes of the configurations and observed salient CC effect in both reward conditions. We found that reward accelerated the implicit learning of contexts in both the early phase and the late phase of the experiment in configurations associated with reward than the ones without reward. The details are as follows.

Firstly, we observed earlier emergence of the CC effect on the Reward condition than Non-reward. In the reward condition, about 178-ms search benefit came forth from the second repetition for repeated displays than for novel displays. This benefit did not show up until five repetitions in the non-reward condition, which was similar to previous studies. Thus, the reward accelerated the contextual learning in the early phase of the experiment. This result probably came about because rewards strengthened the consolidation of spatial context information into memory and improved the participants’ motivation. Secondly, the CC effect was greater in the Reward than in Non-reward condition. Since CC is mostly considered due to the implicit guidance of spatial attention toward the location of the target embedded among the distractor stimuli, we speculated that reward modulated the deployment of attention in the later phase, i.e., guiding attention to the target faster in configurations associated with reward than the ones without reward.

This is a new finding. Although previous studies have suggested that when participants expect a monetary reward they have more negative pre-feedback SPN (Foti and Hajcak, 2012; Fuentemilla et al., 2013; Pornpattananangkul and Nusslock, 2015), there are few studies suggesting that a configuration formed of distractor objects could be implicitly learned and predict feedback information. A recent study found that repeated configurations of distractors could be implicitly associated with information. The emotional modulation of the CC effect was preserved even when affective images were removed from the search display (Zinchenko et al., 2020c). Our results showed that even though there was no overt cue, after several repetitions, observers can take advantage of the learned association between specific configuration and reward, to elicit more negative SPN on the rewarded trial. Moreover, participants could not discriminate repeated from novel contexts in the recognition test immediately following the search task, and most of them reported being unaware of those repeated displays, suggesting that the effects of monetary reward on search were implicit. This might have occurred because seeing a given configuration on the current trial, participants’ visual system accessed an implicit memory trace of the past outcome associated with that specific context, which is sufficient to quickly affect behavior and brain activities on the current trial.

The present study provided more evidence of the debate on what it is that reward is associated with, repeated contexts or repeated target locations, that occur in both repeated and novel displays. In our experiment, the RTs in the Novel-Reward condition were never significantly different from those in the Novel-Non-reward condition for all the seven epochs (see Figure 1B), showing no clear evidence of the association between reward and repeated target location. Conformably, there was no significant difference of SPN amplitude between epoch 1 and epoch 2 for rewarded and non-rewarded new displays, which does not support the overshadowing hypothesis proposed by Sharifian and colleagues (Sharifian et al., 2017). Interestingly, although we found no statistically significant difference in both behavioral and ERP data, we noticed that the SPN amplitude of Novel-Reward was numerically larger than of Novel-Non-reward [*t*(24) = −0.979, *p* = 0.338, *d* = 0.200] and Repeat-Non-reward [*t*(24) = 1.343, *p* = −0.193, *d* = 0.274]. Moreover, consecutive ANOVAs from 400 to 2,000 ms after response onset showed that the interaction between reward and context became significant about 1,000 ms later than the main effect of reward became significant. The possible reason is that reward is associated with both the target position and the overall repeated context. In the repeated context, the expectation of SPN is elicited generally faster, resulting in speeded RTs. In contrast, in the Novel-Reward context, the expectation of reward might be elicited only *post hoc*, i.e., after the target is found in visual search.

Though some previous studies found right hemisphere predominance for SPN, our results showed a left hemisphere preponderance of SPN on reward. One possibility was that we merely used monetary reward but not punishment. In terms of the approach-withdrawal theory by Davidson et al. (1990), the right frontal cortex is involved in withdrawal behaviors, such as punishment, whereas the left frontal lobe is implicated in approach. For the present study, the anticipation of monetary reward activated the left hemisphere, while no punishment activates the right hemisphere, therefore canceling out the inherent right hemisphere domination. Actually, there have been a number of SPN studies using only money that could not find the right hemisphere dominance (Chwilla and Brunia, 1991; Kotani et al., 2003; Ohgami et al., 2004).

The methodology of the present study differs in two ways from that of previous studies that addressed the electrophysiological correlates of CC (Johnson et al., 2007; Schankin and Schubö, 2010; Zinchenko et al., 2020b): the eye movements and the stimulus presentation duration. In previous studies, participants’ eye movements were restricted, since lateralized components analyzed previously required that no eye movements were performed, and the presentation duration was limited to 700 ms to avoid potential eye movements. In contrast, the current study did not limit eye movements, which allowed us to better explore the contextual knowledge that was affected by reward. This could be an important factor since there is some recent evidence that eye movements may trigger either more local or more global processing modes and could thus further influence contextual learning (Zinchenko et al., 2020a). In addition, due to the lack of more electrophysiological components to weigh the validity of the experimental effect, the present study could not further confirm whether the reward could modulate either attention or response selection (Kunar et al., 2007; Schankin and Schubö, 2010) or both.

Another shortcoming of the present experiment is that we only gave a post-cue in reward trials, but not in non-reward trials. One might argue that participants’ better memory for repeated displays with reward is not due to the reward signal in our study but more generally due to the presence vs. absence of a post-cue. Combined with previous behavioral studies to come up with our behavioral data, the post-cue in reward trials enhanced the motivation in the present study. For example, Wachter et al. investigated the influence of feedback on the efficacy of implicit learning, and they found that positive feedback fortified learning more than negative feedback. In our experiment, if we had given feedback in our non-reward trials, which had been relatively “negative” as compared with reward feedbacks, we would have expected to see similar results as in the present study. In addition, in current studies that are in progress, we have amended this deficiency by giving post-cues in every trial, and we found the RTs for reward repeated displays were still shorter than for non-reward repeated displays, so we think this design weakness will not influence the conclusion we made.

The last point we want to discuss is the recognition test results. As we mentioned previously, Geyer et al. (2010, 2012) have reported that some repeated displays are explicitly remembered with an extended recognition test or more powerful analysis methods (Smyth and Shanks, 2008; Schlagbauer et al., 2012; Vadillo et al., 2016). In our experiment, although the hit rate for Novel-Reward was not statistically significantly higher than the FA rate, numerically, they were much higher. Thus, it seems to require more investigations about the implicit or explicit nature of the CC effect.

In conclusion, our findings provide evidence that rewarded repeated contexts are searched more efficiently in the CC paradigm. Incidentally learned association between reward and contexts accelerates attentional guidance, which was observed only in repeated contexts. This association is reflected by larger SPN following in rewarded configurations. Our study adds to the growing research on reward-driven attention selection.

## Materials and Methods

### Participants

Twenty-seven undergraduate students participated in the current study. One participant was excluded from analyses because of poor-quality EEG recordings, and one was excluded for excessive error rate (>20%). This gave rise to a final sample of 25 participants (14 females and 11 males, aged between 18 and 25 years, mean age = 20.5 years). All participants were right-handed, reported normal or corrected-to-normal vision, and were naïve as to the purpose of the experiment. All procedures and methods were conducted in accordance with guidelines approved by the Research Ethics Committee of Tianjin Normal University in China. Written informed consent was obtained from all participants.

### Procedure

We used a visual search task similar to that of Chun and Jiang (1998), but we added additional feedback after the participants’ response. This feedback informed observers whether they got a monetary reward for their current performance.

**FIGURE 4:**
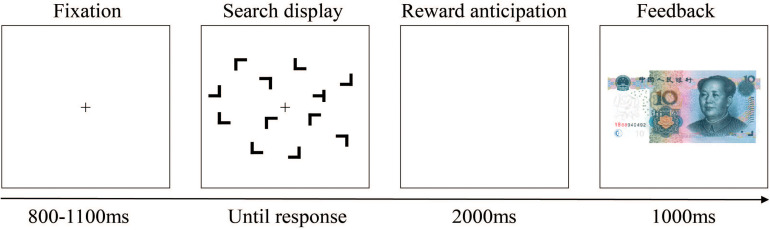
Schematic representation of a single trial. After the fixation, a search display was presented. After participants’ searching and pressing the keyboard, a blank of 2,000 ms arose, followed by the feedback suggesting that whether there was a reward (with a paper money image) or not (without image; i.e., there was a blank screen) in this trial. For this trial, there was a reward.

Participants were seated comfortably in a dimly lit and sound-attenuating chamber and approximately 60 cm away from a computer screen. Each trial began with the presentation of a black fixation cross at the center of the color monitor; 800–1,100 ms later, the search display within an invisible 8 × 6 grid that subtended approximately 12.4° × 8.9° in visual angle appeared. Each search display consisted of 12 black stimuli on the gray background. The size of the stimuli in this experiment was about 1.5° × 1.4° in visual angle. Participants were instructed to respond as quickly and accurately as possible to the orientation of the target “T,” which rotated 90° to the right or to the left amidst 11 distractor stimulus “L’s” presented randomly in one of four rotations (0°, 90°, 180°, and 270°), by pressing corresponding keys on the keyboard with their left or right index finger. Displays were visible until a response was made or until 3,000 ms had elapsed. Following observers’ search task response and another blank interval of 2,000 ms, the feedback stimulus appeared, and an image of 10 CNY paper money meant participants gain 0.1 *yuan* monetary reward in this trial, while a blank screen meant that they do not get money. Feedback stayed on the screen for 1,000 ms, and then the next trial started. An example trial is illustrated in Figure 4.

Unbeknownst to participants, eight of the contexts were repeated across blocks throughout the experiment (eight distinct associations between a specific layout of items in the display and a corresponding target location), called Repeated configurations. Novel configurations were randomly generated at the beginning of each trial and never repeated, so they provided no predictive information regarding the target location. To prevent the target probability learning, 16 locations were chosen from 48 locations as target locations in advance. Eight of the 16 target locations were randomly assigned to repeated displays, and the other eight locations were assigned to novel configurations. Repeated contexts are intermixed with Novel configurations within blocks. Thus, one block contained 16 trials. Each repeated context has a unique target position. For each repeated context, the locations of target and distractors held consistency, but their orientations were randomized across blocks throughout the entire experiment. On the other hand, half of the repeated configuration trials were followed by a reward feedback as well as novel trials, while the other half was followed by a blank screen feedback. Therefore, trials were classified into four conditions: Repeated-Reward, Repeated-Non-reward, Novel-Reward, and Novel-Non-reward. For repeated configurations, four configurations were associated with Reward feedback, while four were associated with Non-reward feedback. For novel configurations, half of the contexts with four specific target locations were associated with Reward feedback, and the other half with the other four specific target locations were associated with Non-reward feedback. Participants performed 28 blocks of 16 trials each, yielding a total of 448 trials (112 trials for each condition). Thus, the eight repeated contexts would appear 28 times dispersed throughout the whole experiment, and each context was exposed only once in a block. To improve the statistical power, 28 blocks were collapsed into seven epochs. Before the formal experiment, there was a practice with 16 trials to familiarize the participants with the procedure. It is worth noting that only the new displays were included in the practice.

In addition, to equate target location repetition effects between the two types of displays, the target appeared equally often at each of 16 possible locations throughout the experiment. Furthermore, for both repeated and novel displays, the target was equally likely to appear in any of the four display quadrants, and the item density was kept constant across the four display quadrants (each quadrant contained three items). During the experiment, reward values (Reward and Non-reward) were associated with specific target locations for novel configurations as well as repeated configurations. Each condition consistently corresponds to particular target locations.

At the end of experiment, a display recognition test was carried out. Participants were informed of the repetition of some of the search displays throughout the experiment by being shown a screen containing an instruction. Participants initiated the presentation of another 16 trials, which include the eight repeated configurations and eight newly generated displays, and they decided (forced choice) via keyboard responses whether a particular display had been shown previously or not.

### Recording

Electroencephalography was recorded from 64 sintered Ag/AgCI electrodes mounted in an elastic cap according to the extended 10/20 system. Software-linked mastoids (M1, M2) served as a common reference. Horizontal electrooculogram (EOG) was recorded from a pair of electrodes placed at the outer canthi of the eyes to detect horizontal eye movements. Vertical EOG was recorded from two electrodes placed on the left infraorbital and supraorbital areas to monitor vertical eye movements and blinks. All electrode impedances were kept below 7 kΩ. The EEG and EOG were amplified and digitalized using an ANT Neuro amplifier with a frequency of 500 Hz and a low-pass filter at 100 Hz in DC acquisition mode. Trials in which EEG or EOG voltages exceeded a threshold of 100 mV during the recording epoch were excluded from further analysis. The trials containing muscular activity were also removed. EEG data were processed using EEGLAB (Ohgami et al., 2004), running on MATLAB. After these procedures, the number of trials average ranged from 80 to 110 of each condition for testing the effect of current outcome on the SPN. Data were filtered off-line by a band-pass filter of 0.1–30 Hz to run an independent component analysis (ICA) for eye movement correction.

### Data Analysis

Reaction times were measured as the time between onset of the search display and response. Pressing the wrong button, pressing the button too quickly (<200 ms), and pressing it too slowly (>3,000 ms) were defined as errors. And the RTs exceeded ± 3 standard deviations will be discarded from analyses. A total of 1.2% of data was removed. Repeated-measures ANOVAs with three factors (configuration × reward condition × epoch) were conducted on behavioral data for accuracy and reaction times. In addition, the Bayes factors analysis was carried out for results biased toward the null hypothesis. A Bayes factor (null/alternative) value greater than 3 is considered to be “substantial” evidence for the null hypothesis.

Event-related potentials were calculated time-locked to the onset of the response, with segments extending from -200 ms before response onset until 3,100 ms afterward. Note that after 2,000 ms of response onset, the feedback appeared. And during this 2,000-ms interval, participants’ conjecturing about whether there was a reward or not in this trial would eject an SPN. Mean SPN amplitudes over the 200-ms interval preceding the feedback stimulus (i.e., 1,800–2,000 ms after the response onset) were calculated at central (C1/2, C3/4), centroparietal (CP3/4), and parietal (P1/2, P5/6) regions. The activity from −200 to 0 ms served as the baseline. The data were subjected to an ANOVA using within-subjects factors of Configuration (repeated, novel), Reward (reward, non-reward), Region (central, centroparietal, parietal), and Hemisphere (left, right). Off-line epochs were computed from 200 ms proceeding to 3,100 ms following the button press.

A repeated-measures ANOVA design with four factors (Configuration × Reward condition × Region × Hemisphere) was conducted to assess SPN. To further analyze the SPN variation, the SPN sustained negativity was analyzed every 200-ms interval from 400 to 2,000 ms to the onset of response activation, for a total of eight time-windows. Each time window conducted a repeated-measures ANOVA analysis with four factors (Configuration × Reward condition × Region × Hemisphere).

## Data Availability Statement

The original contributions presented in the study are included in the article/supplementary material, further inquiries can be directed to the corresponding author.

## Ethics Statement

The studies involving human participants were reviewed and approved by the Research Ethics Committee of Tianjin Normal University. The patients/participants provided their written informed consent to participate in this study.

## Author Contributions

GZ and QZ designed and implemented the experiments. QZ and SL collected the data. JM and QZ analyzed the data. QZ created the initial draft of the manuscript. ST and GZ revised the manuscript. All authors reviewed the manuscript.

## Conflict of Interest

The authors declare that the research was conducted in the absence of any commercial or financial relationships that could be construed as a potential conflict of interest.
